# A Novel Partitivirus That Confer Hypovirulence to the Plant Pathogenic Fungus *Colletotrichum liriopes*

**DOI:** 10.3389/fmicb.2021.653809

**Published:** 2021-06-22

**Authors:** Jun Zi Zhu, Jun Guo, Zhao Hu, Xu Tong Zhang, Xiao Gang Li, Jie Zhong

**Affiliations:** ^1^Hunan Engineering Research Center of Agricultural Pest Early Warning and Control, Hunan Agricultural University, Changsha, China; ^2^Hunan Provincial Key Laboratory for Biology and Control of Plant Diseases and Insect Pests, Hunan Agricultural University, Changsha, China

**Keywords:** partitivirus, *Colletotrichum liriopes*, hypovirulence, dsRNA, Colletotrichum liriopes partitivirus 1

## Abstract

Here, we report a novel double-stranded RNA virus designated Colletotrichum liriopes partitivirus 1 (ClPV1) from the plant pathogenic fungus *C. liriopes.* ClPV1 genome has two double stranded RNAs (dsRNAs), named as dsRNA 1 and dsRNA 2, which in the lengths of 1,807 and 1,706 bp, respectively. The dsRNA 1 and dsRNA 2 encoded proteins showing significant amino acid (aa) sequence identity to the RNA-dependent RNA polymerase (RdRp) and coat protein (CP) of partitiviruses, respectively. Phylogenetic analysis using the aa sequences of RdRp and CP indicated that ClPV1 was grouped to members of the putative Epsilonpartitivirus genus in the *Partitiviridae* family. Spherical viral particles in approximately 35 nm in diameter and packaging the ClPV1 genome were isolated. Virus elimination and virus transfection with purified viral particles, and biological comparison revealed that ClPV1 could reduce the virulence and conidia production of *C. liriopes*. To the best of our knowledge, this is the first report of mycovirus in *C. liriopes* fungus.

## Introduction

Mycoviruses or fungal viruses are commonly found that can infect almost all major taxa of fungi including plant-pathogenic fungi and replicate in these organisms (Ghabrial and Suzuki, 2009; Xie and Jiang, 2014; Ghabrial et al., 2015). Mycoviruses with genomes of double stranded RNA (dsRNA) can be currently classified into or affiliated to families including *Totiviridae*, *Partitivirida*e, *Chysovirida*e, *Quadriviridae*, *Endornaviridae*, *Megabirnaviridae*, *Reoviridae*, *Amalgaviridae*, *Curvulaviridae*, *Polymycoviridae*, and a kingdom, *Orthornavirae* (Ghabrial and Suzuki, 2009; Xie and Jiang, 2014; Ghabrial et al., 2015; Sato et al., 2018).

Viruses in the family *Partitiviridae* have been shown to infect fungi, plants, and protozoa (Nibert et al., 2014; Vainio et al., 2018). At present, viruses in this family were divided into five genera: *Alphapartitivirus*, *Betapartitivirus*, *Gammapartitivirus*, *Deltapartitivirus*, and *Cryspovirus* (Nibert et al., 2014; Vainio et al., 2018). Recently, two new genera, named as Epsilonpartitivirus and Zetapartitivirus have been proposed (Nerva et al., 2017; Jiang et al., 2019). In generally, the genome of each partitivirus consist of two linear, dsRNA segments, which have the lengths ranging from 1.4 to 2.4 kbp and are encapsidated in rigid, spherical virus particles with diameters of approximately 25–40 nm. Each of the dsRNA genome segments contains an open reading frame (ORF), with the larger segment encodes the RNA-dependent RNA polymerase (RdRp) and the smaller one encodes the capsid protein (CP), respectively, Nibert et al. (2014) and Vainio et al. (2018).

Although the vast majority of mycoviruses are associated with latent infections, however, some mycoviruses can cause clearly phenotype alterations, such as in growth, sporulation, pigmentation, and virulence that leading to hypovirulence and debilitation. Moreover, a number of mycoviruses were reported to have beneficial effects on the host fungi, such as the increase of virulence (hypervirulence) in plant pathogenic fungi or oomycetes (Jian et al., 1997; Ahn and Lee, 2001), the enhancement of competitive ability via producing killer proteins in some yeasts (Park et al., 1996; Schmitt and Breinig, 2006), and the improvement of heat tolerance in plant conferred by the symbiotic host fungus (Márquez et al., 2007). During the last few decades, in view of the potential biological usefulness of mycovirus-mediated hypovirulence to control fungal diseases, as has been exampled by the successfully use of Cryphonectria hypovirus 1 to control chest blight disease in Europe (Nuss, 1992; Xie and Jiang, 2014; Ghabrial et al., 2015), many researchers were inspired to hunt mycovirus for useful virocontrol agents. In addition, some mycoviruses can increase the virulence of the host fungus, which could also be usefully for characterization of the molecular mechanism of virulence regulation in fungi (Jian et al., 1997, 1998; Lau et al., 2018; Okada et al., 2018). Thus, many mycoviruses were subsequently found, largely expanding our knowledge on mycoviral diversity, ecology, and evolution.

*Colletotrichum* spp. are considered as an economically significant groups of plant pathogens that cause anthracnose disease in a variety of plant species worldwide (Cannon et al., 2012). *Colletotrichum liriopes* is a species of the *Colletotrichum* genus causing leaf anthracnose in many plants. *C. liriopes* was first reported as the pathogen fungus on *Liriope muscari* in Mexico (Damm et al., 2009). In recent years, the host rang and distribution area of this fungus have gradually expanded, such as in *Liriope spicata* (Chen et al., 2019), *L. cymbidiomorpha* (Yang et al., 2020), *Rohdea japonica* (Kwon and Kim, 2013; Trigiano et al., 2018), *Bletilla ochracea* (Tao et al., 2013), and *Ophiopogon japonicus* (Wang and Wang, 2021). Therefore, this species might brought a threat in agricultural production. Up to date, a number of mycoviruses have been isolated and identified from the *Colletotrichum* fungus. An unidentified dsRNA virus possessing isometric viral particles was previously reported in *C. gloeosporioides* (Figueirêdo et al., 2012). Later, a virus in the family *Partitiviridae* was characterized in the fungus *C. acutatum* (Zhong et al., 2014). Some endophytic or phytopathogenic strains of the *Colletotrichum* genus were screened for other mycoviruses or dsRNA elements (Marzano et al., 2016; Rosseto et al., 2016). Zhong et al. (2016) reported a novel chrysovirus with three dsRNA genome segments from the fungus *C. gloeosporioides.* Campo et al. (2016) reported a non-segmented dsRNA mycovirus in the phytopathogenic fungus *C. higginsianum*. Jia et al. (2017) reported a mycovirus containing filamentous viral particles from the *C. camelliae* that was belonging to the proposed family *Polymycoviridae*. Moreover, a hepta-segmented tentative chrysovirus that has the ability to confer hypovirulence was reported in *C. fructicola* (Zhai et al., 2018). Recently, a mycoviruses that was distinct from members of the family *Partitiviridae* (Wang et al., 2019), as well as an ourmia-like virus, were isolated and identified from the *C. gloeosporioides* (Guo et al., 2019).

In this study, we characterized a novel mycovirus, namely Colletotrichum liriopes partitivirus 1 (ClPV1), from a *C. liriopes* strain Cl-B-2. Viral genome organization and phylogeny analysis indicate that this virus was a new member of the family *Partitiviridae.* Further, we also determine the effects of viral infection on the *C. liriopes* strains.

## Materials and Methods

### Fungal Isolates, Growth Conditions

*Colletotrichum liriopes* strains Cl-B-2 and SJM3-2 were originally isolated from the *Paris polyphylla* and *Pachysandra terminalis* plants that were infected by anthracnose disease, respectively, in Hunan province of China. The Cl-B-2-P1 was a derivative strain of the Cl-B-2 obtained by protoplast regeneration. The SJM3-2-T5 was a derivative strain transfected with viral particles of ClPV1. All strains were grown on potato dextrose agar (PDA; potato, glucose, agarose) at 27°C. For dsRNA or total RNA extraction, mycelial plugs were cultured on potato dextrose (PD) broth (potato, glucose) liquid medium at 27°C, with an orbital shaker at 110 rpm for 4 to 7 days.

### DsRNA Extraction and Purification

Double stranded RNAs were extracted from fungal mycelium using CF-11 cellulose (Sigma, St. Louis, MO, United States) column chromatography with the methods described by Morris and Dodds (1979) with modifications. DNA and ssRNA contaminants were eliminated by digestion with RNase-free DNase I and S1 nuclease (Takara, Dalian, China), respectively. The extracted dsRNAs were fractionated by agarose gel (1%, w/v) electrophoresis and visualized under an AlphaImager HP gel imaging system (ProteinSimple, Silicon Valley, CA, United States) after being stained with 0.1 mg/mL ethidium bromide.

### cDNA Cloning and Sequencing

The dsRNAs were purified and used as a template for cDNA synthesis. The cDNA library was constructed using random hexadeoxynucleotide primers (Takara, Dalian, China) based on the methods described previously (Zhong et al., 2016). Internal gap regions of the viral genome were filled by reverse transcription-PCR (RT-PCR) amplification through RT-PCR using sequence-specific primers designed based on obtained sequences. To obtain the terminal sequence of each of the dsRNAs, a ligase-mediated terminal amplification method was used. All of the amplified DNA fragments were purified, cloned and Sanger sequenced. Every base was determined by sequencing at least three independent overlapping clones.

### Sequence Analysis

Potential ORFs in each full-length cDNA sequence were deduced and their homologous amino acid (aa) sequences were searched in NCBI by ORF Finder and BLASTp programs, respectively. Multiple sequence alignment was carried out using the CLUSTALX 1.8 program (Thompson et al., 1997). Phylogenetic analysis was carried out with the maximum likelihood or neighbor-joining method in MEGA 7 programs (Kumar et al., 2016). Bootstrap values supporting the phylogenetic tree were calculated after 1,000 re-samplings.

### Curing of Virus From Strain Cl-B-2

We performed the protoplast regeneration method to eliminate ClPV1 from its host fungus. Protoplast preparation was made using the method as described by Lau et al. (2018). The regenerated isolates were individually transferred to fresh PDA and subjected for virus detection by dsRNA extraction and RT-PCR, using the specific primers (ClPV1-F: 5′-CAAAGGAGAAGTTAT CGGAAGC-3′/ClPV1-R: 5′-AAGGTCAGCGGACAA GGATA-3′) designed based on the RdRp encoding sequence of ClPV1.

### Vertical Transmission of ClPV1

Single-spore isolation and virus detection were carried out for evaluating the transmission efficiency of virus ClPV1. Conidia were produced on PDA plate and single-spore isolates were obtained by picking the conidia individually into fresh PDA plates. The presence of virus in the single-conidian isolates was confirmed by RT-PCR detection.

### Purification of Virus Particles

Virus particles were purified from mycelial of *C. liriopes* strain Cl-B-2 according to the procedure described previously by Chiba et al. (2009). Briefly, *C. liriopes* was grown on PD for 7 days. Approximately 50 g of mycelia were harvested and ground to fine powder with liquid nitrogen. The powder was mixed with 150 mL phosphate buffer (0.1M sodium phosphate, pH 7.0 containing 2% Triton X-100). Then, the suspension was centrifuged at 10,000 × *g* for 30 min to remove the hyphal cell debris. The supernatant was transferred to centrifugation at 100,000 × *g* under 4∘C for 2 h. The resultant pellet was resuspended in 0.1M phosphate buffer and subsequently fractioned with 20–50% (w/w) sucrose gradients for 2.5 h at 100,000 × *g* in a Beckman SW55 rotor. The fractions containing particles were further precipitated by ultracentrifugation at 100,000 × *g*. The pellet was resuspended in 100 μl of 0.01M sodium phosphate buffer, pH 7.0.

A drop of purified virus suspension was stained negatively with 1% uranyl acetate and observed under a transmission electron microscope (TEM). The dsRNAs from the viral particles were extracted with phenol chloroform isoamyl alcohol and detected using 1% agarose gel electrophoresis. The purified virus particles were subjected for sodium dodecyl sulfate (SDS)-polyacrylamide (12%) gel electrophoresis and stained with Coomassie brilliant blue R250.

### Transfection With ClPV1 Particles

The virus-free *C. liriopes* strain SJM3-2 was used as a virus recipient in the transfection experiment. Protoplasts of the recipient were prepared using the method described previously (Kanematsu et al., 2004), and subjected for protoplast transfection in the presence of 50% (wt/vol) polyethylene glycol 6000 as previously described (Jia et al., 2017). About 2 × 10^7^ protoplasts were mixed with purified virus particles. Transfected protoplasts were spread onto regeneration media (0.7 mol/L sucrose, 0.5 g/L yeast extract, 15 g/L agar) plates for colony regeneration at 27°C. The putative transfected regenerated isolates were selected and sub-cultured on new PDA plates. The presence of virus infection was tested by dsRNA extraction and RT-PCR amplification using viral specific primer pairs.

### Biological Assessment

Morphology and growth rates were assessed by culturing mycelial plugs, collected from 4-day-old actively growing plates, on PDA for 3–7 days at 27°C in the dark. Mycelial growth rates were tested by measuring the diameters of each colony, with each strain has three replicates.

For virulence assays, mycelial plugs of each strain were inoculated on detached fruits of apple (*Malus pumila* Mill.) as described previously (Xie et al., 2016). All the inoculations were maintained in humid container at 27°C. The experiments were conducted twice with each strain has five replicates. The developed lesions were photographed and measured after 7 days of inoculation.

Data were analyzed by the SPSS program. Differences between the treatments were compared by using one-way ANOVA and S-N-K(s) test, *P* < 0.05 was considered to indicate statistical significance.

## Results

### DsRNAs in *C. liriopes*

**FIGURE 1:**
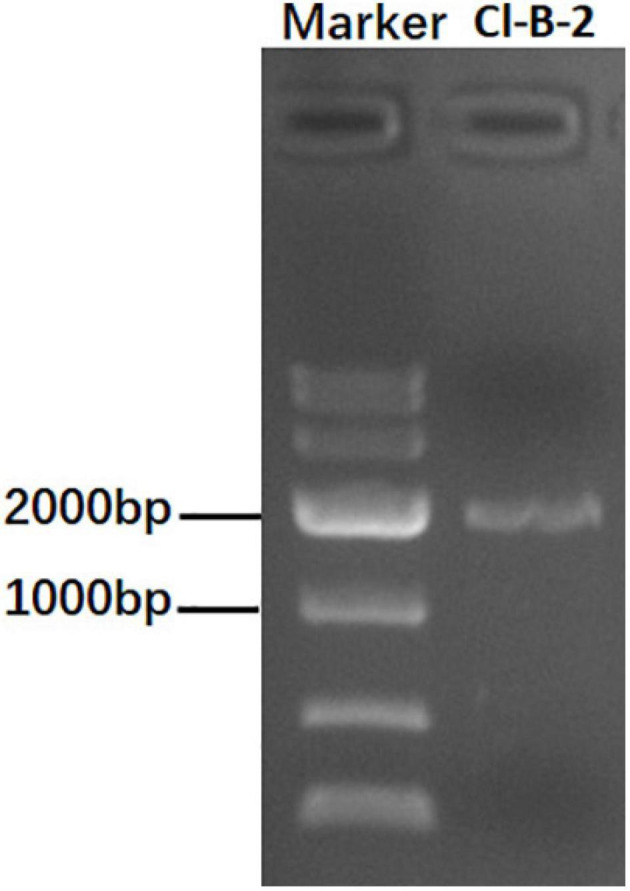
Agarose gel electrophoresis of dsRNA extracted from the *Colletotrichum liriopes* strain Cl-B-2. The dsRNA extractions were treated with S1 nuclease and DNase I before being electrophoresed on 1% agar gel.

When screened by a method using cellulose, a *C. liriopes* strain Cl-B-2 was found to be dsRNA-positive. After being treated with DNase I and S1 nuclease, a dsRNA band, in the size of approximately 2 kbp was observed on a 1% agarose gel (Figure 1). The subsequent experiments revealed that the dsRNA band was actually comprised of two dsRNAs that we termed as dsRNA 1 and dsRNA 2.

### Nucleotide Sequence and Genome Organization of ClPV1

**FIGURE 2:**
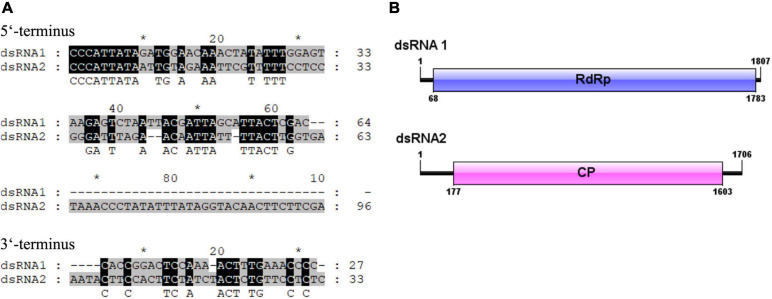
**(A)** Terminal sequences between dsRNA 1 and dsRNA 2 were aligned and the identical sequences were indicated with reverse highlighting. **(B)** Schematic representation of the genome organization of virus Colletotrichum liriopes partitivirus 1 (ClPV1). ClPV1 contained two dsRNAs, dsRNA 1, and dsRNA 2, which encoded a putative RNA-dependent RNA polymerase (RdRp) and a coat protein (CP), respectively. The open reading frames (ORFs) were indicated by open bars and the 5′ and 3′ untranslated regions were indicated by single lines.

The full-length cDNA sequences of the two dsRNA segments were determined by cloning and assembling of cDNAs obtained from random RT-PCR and rapid cDNA ends amplification protocol. The resulting full-length sequences of the two dsRNAs, dsRNA 1 and dsRNA 2, were in the length of 1,807 and 1,706 bp, respectively. The cDNA sequences of each of the dsRNA segments were predicted to include a single ORF, named ORF1 and ORF2, respectively. The 5′ and 3′-untranslated regions (UTRs) were 64 and 27 bp in the dsRNA1, and 176 and 33 bp in the dsRNA 2, respectively. The 5′-terminus between the two dsRNAs were conserved containing the conserved stretch “CCCATTATA.” Conserved terminal sequences might be involved in recognition and replication of the viral dsRNAs. The 3′ UTRs between the dsRNA 1 and dsRNA 2 were shorter and less conserved (Figure 2A). In view of that the dsRNA 1 and dsRNA 2 showed similarity to the RdRp and CP genes of partitiviruses, the dsRNA 1 and dsRNA 2 might represent the genome of a partitivirus, which we designed it as ClPV1. The putative genome organization of ClPV1 was showed in Figure 2B. The full-length cDNA sequences for the dsRNA 1 and dsRNA 2 were deposited in GenBank with accession numbers MW291533 and MW291532, respectively.

**FIGURE 3:**
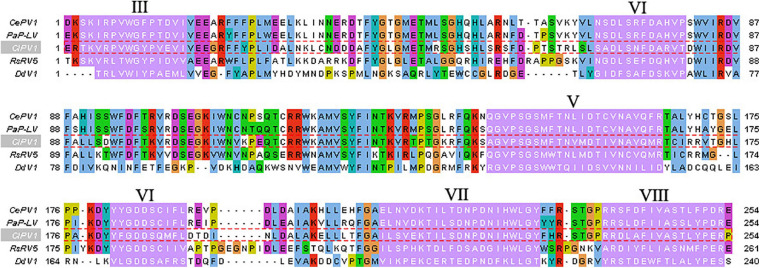
Multiple sequence alignment of the aa sequences of the RdRp encoded by ClPV1. Conserved motifs III–VIII were showed by Roman numerals. The abbreviations of virus names are as follows: CePV1, *Colletotrichum eremochloae* partitivirus 1 (AZT88590.1); PaP-LV, *Penicillium aurantiogriseum* partiti-like virus (YP_009182157.1); RsPV5, *Rhizoctonia solani* dsRNA virus 5 (AVP26802.1); and DdV1, Discula destructiva virus 1 (NP_116716.1).

Colletotrichum liriopes partitivirus 1 ORF1 was predicted to encode a polypeptide comprised of 571 aa residues with a calculated molecular mass of 64.91 kDa. Homology searches revealed that the 64.91 kDa protein was similar to the RdRp of partitiviruses, including Metarhizium brunneum partitivirus 1 (MbPV1, accession no: QHB49873.1, Query cover: 99%; *E* value: 0; identity: 65.21%), Plasmopara viticola lesion associated Partiti-like 2 (PvlaPL2, accession no: QNQ74068.1, Query cover: 99%; *E* value: 0; identity: 57.09%), Colletotrichum eremochloae partitivirus 1 (CePV1, accession no: AZT88590.1, Query cover: 99%; *E* value: 0; identity: 59.30%), Penicillium aurantiogriseum partiti-like virus (accession no: YP_009182157.1; *E* value: 0; Query cover: 99%; identity: 58.42%), Penicillium aurantiogriseum partitivirus 1-cp (accession no: AZT88590.1; Query cover: 99%; *E* value: 0; identity: 59.30%), et al. When searched by conserved domain database search and multiple protein alignment, conserved motifs (Motif III to Motif VIII) which were also present in and characteristic of RdRp sequences of other members of the *Partitiviridae* family, were detected in the ClPV1 encoded RdRp (Figure 3).

The ORF2 in dsRNA 2 potentially encoded a protein of 498 aa, with a molecular mass of 55.51 kDa. BLASTp search revealed that the ClPV1 ORF2 had significant aa sequence identity, ranging from 30.36 to 58.37%, to the CP of the family *Partitiviridae*. The MbPV1 showed the best matching, with aa identity of 50.10% (*E* value: 1e ^–173^; Query cover: 97%), followed by PvlaPL2 and CePV1.

**FIGURE 4:**
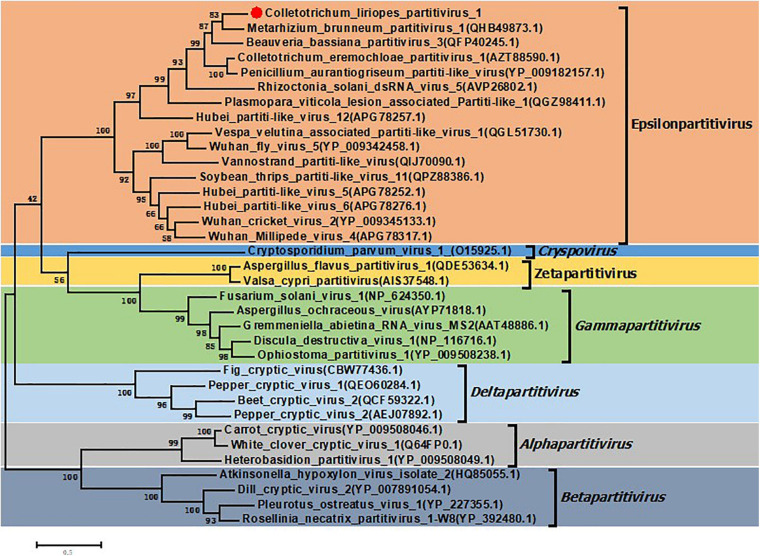
Phylogenetic analysis of ClPV1. Phylogenetic trees were generated based on the amino acid sequences of the RdRp of ClPV1 and other selected partitiviruses, respectively. The phylogenetic tree was constructed using maximum likelihood method with bootstrapping analysis of 1000 replicates. ClPV1 was indicated in the phylogenetic tree. The scale bars mean the estimated number of substitutions per 100 amino acids.

In order to clarify the evolution status of the ClPV1, phylogenetic analysis using the aa sequences of RdRp and CP were performed. The RdRp-based phylogenetic tree showed that ClPV1 was most closely related to relative viruses, such as MbPV1, in an unclassified cluster, which might be affiliated to the new putative genus Epsilonpartitivirus in the family *Partitiviridae* (Figure 4). In addition, phylogenetic analysis with the ClPV1 CP also indicated a similar taxonomic status in the family *Partitiviridae* (Supplementary Figure 1).

### Characterization of Viral Particles

**FIGURE 5:**
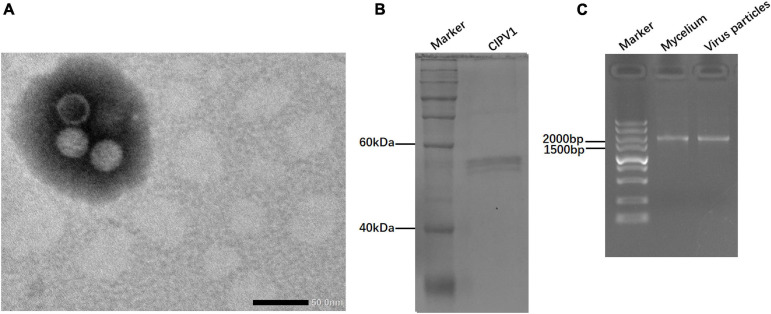
Virus particles isolated from the mycelia of *C. liriopes* strain Cl-B-2. **(A)** Isometric, non-enveloped viral particles with diameter of 35 nm were observed by transmission electron microscope (TEM). **(B)** SDS-PAGE electrophoresis (12%) analysis of the purified virus particles showing the protein band of coat protein. **(C)** Agarose gel electrophoresis of dsRNA extracted from the mycelia and viral particles, respectively.

Virus particles of ClPV1 were purified by sucrose density-gradient centrifugation. Under TEM, viral particles were isometric in shape, with an average diameter of 35 nm (Figure 5A). SDSPAGE electrophoresis indicated a single protein band of 55 kDa, which was consistent with the predicted protein encoded by ORF2 (Figure 5B). In addition, the viral particles had dsRNAs with the same size as those purified from the mycelia of *C. liriopes* strain Cl-B-2 (Figure 5C).

### Vertical Transmission of ClPV1

In order to investigate the transmission efficiency of ClPV1, a total of seventy single-spore isolates were obtained. Detection of ClPV1 was conducted by RT-PCR using the specific primer pair. Results showed that all of the fungal progenies harbored the ClPV1, indicating that ClPV1 in the host fungus could be vertically transmitted, with a high efficiency, to the single-conidium progenies (Supplementary Figure 2).

### Virus Elimination, Viral Particles Transfection, and Effects of ClPV1 Infection on Growth and Pathogenicity of *C. liriopes*

**FIGURE 6:**
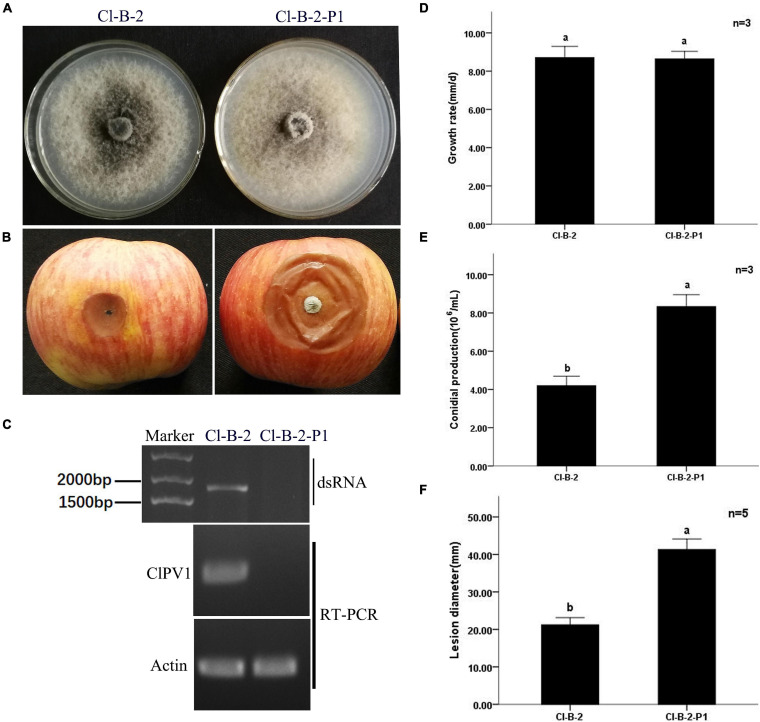
Virus curing of ClPV1 from *C. liriopes* strain Cl-B-2. **(A)** Colony morphology of *C. liriopes* strain Cl-B-2 and a virus free derivative of Cl-B-2-P1. Fungal strains were grown on PDA at 27∘C for 7 days. **(B)** Pathogenicity analysis of the Cl-B-2 and Cl-B-2-P1 strains. The symptoms were observed 14 days post inoculation. **(C)** Detection of ClPV1 in strains Cl-B-2 and Cl-B-2-P1 using dsRNA extraction and RT-PCR methods, with the actin gene of *C. liriopes* served as an internal control. **(D)** Comparison of the average radial mycelial growth rates between the two fungal strains after being cultured at 27∘C on PDA for 7 days. **(E)** Comparison of the conidial production of the two fungal strains on PD for 5 days. **(F)** Average lesion length on apple fruits caused by strains Cl-B-2 and Cl-B-2-P1. The a, b indicate a significant difference at the *P* < 0.05 level of confidence.

To determine the effects of ClPV1 on the fungal host phenotype, we conducted the protoplast regeneration for virus elimination. Strains of single colony were collected and confirmed to be virus free using dsRNA extraction and RT-PCR detection with specific primers. On PDA plates, no significant difference in mycelium growth rate between the virus-free Cl-B-2-P1 and its paternal virus-containing Cl-B-2 was observed. Colony morphologies between these two isogenic strains were similar with the exception that the virus infected Cl-B-2 has a larger area in black pigments. In addition, there was significant difference in conidia production between the virus infected and virus free strains, with the virus free strain Cl-B-2-P1 has more conidia produced (8.33 × 10^6/^mL vs. 4.21 × 10^6^/mL). Virulence assays on detached apple fruits were conducted. After 14 days, the average lesion areas caused by Cl-B-2-P1 (41.3 mm) were larger than those caused by Cl-B-2 (21.2 mm), indicating that ClPV1 might confer hypovirulence to its fungal host *C. liriopes* strain Cl-B-2 (Figure 6).

**FIGURE 7:**
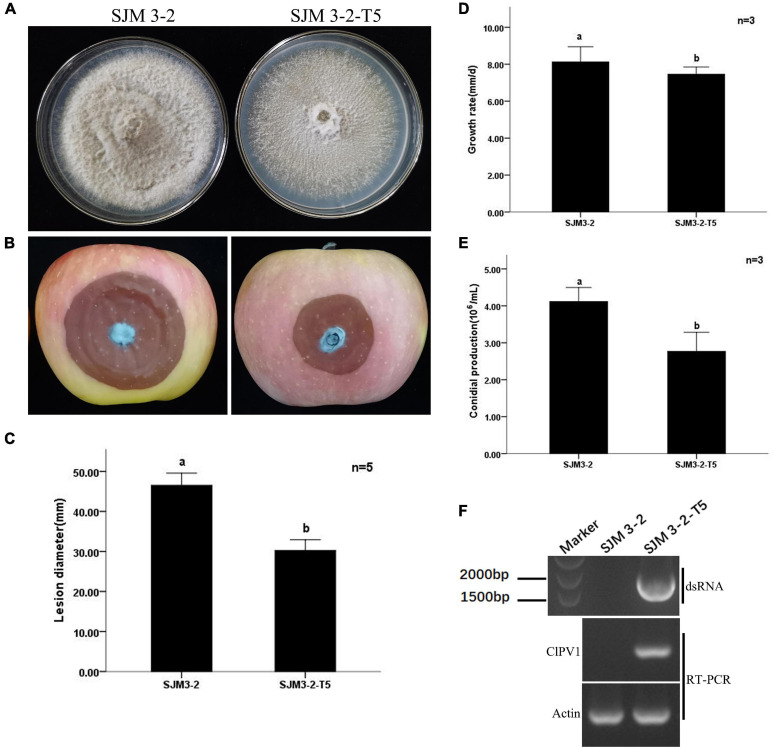
Transfection of protoplasts of a virulent *C. liriopes* strain SJM3-2 with purified virus particles of ClPV1. **(A)** Colony morphology of strains SJM3-2 and a ClPV1 transfected derivative strain SJM 3-2-T5 on PDA at 27°C for 7 days. **(B)** Virulence test on apple fruits by inoculating with SJM 3-2 and SJM 3-2-T5. Symptoms were observed after 14 days of inoculation. **(C)** Average lesion lengths caused by SJM 3-2 and SJM 3-2-T5. **(D)** Comparison of the average radial mycelial growth rates between SJM 3-2 and SJM 3-2-T5 on PDA at 27∘C for 7 days. **(E)** Comparison of the conidial production of the two strains on PD at 27∘C for 5 days. **(F)** Virus detection in SJM 3-2 and SJM 3-2-T5 using dsRNA extraction and RT-PCR. The a, b indicate a significant difference at the *P* < 0.05 level of confidence.

To determine whether ClPV1 could cause phenotype change in other *C. liriopes* strain. We transfected the protoplasts of a wild type, virus-free *C. liriopes* strain SJM3-2, using the purified viral particles and a PEG-mediated protoplast transfection method. A derivative virus-transfected strain, SJM 3-2-T5, was selected for biological comparison. Compared to the isogenic virus-free strain SJM3-2, SJM 3-2-T5 showed a slightly slower growth rate and a lesser pigmentation on PDA plates. Moreover, the virus infected SJM 3-2-T5 had a lower conidia production compared to the virus free SJM3-2 (4.20 × 10^6^/mL vs. 2.71 × 10^6^/mL). The lesion diameter caused by SJM 3-2-T5 on apple fruits was significantly smaller than that caused by SJM3-2 (30.3 mm vs. 46.5 mm; Figure 7). Therefore, except the host fungus, ClPV1 could induce hypovirulence on other *C. liriopes* strain.

## Discussion

Screening of more mycoviruses might, in one hand, expand our understanding of viral diversity and evolution, and in other hand, provide more resource with valuable biological potential (Xie and Jiang, 2014). In this study, we reported the discovery of a novel mycovirus, ClPV1, from the plant pathogenic fungus *C. liriopes.* ClPV1 has features typical of partitivirus, with genome comprised of two dsRNA segments, the dsRNA 1 and dsRNA 2, encapsidated with isometric particles with 35 nm in diameter. Besides, the 5′-UTRs of the two dsRNA segments showed conserved terminal stretch and similar stem-loop structure (Supplementary Figure 3), which might be associated with virus replication and RdRp recognition. The two dsRNA segments of ClPV1 were predicted to contain a single ORF encoding proteins shared significant aa identities to the RdRp and CP, respectively, of the partitiviruses. Phylogenetic analysis using the aa sequences of RdRp and CP indicated that ClPV1 was clustered with members of the putative genus Epsilonpartitivirus in the family *Partitiviridae.* Thus, according to genome organization, viral particle morphology, complete nucleotide sequences, and phylogenetic analysis, ClPV1 was a new member of the putative genus Epsilonpartitivirus in the family *Partitiviridae* in the family *Partitiviridae.* To the best of our knowledge, this was the first report of mycovirus in *C. liriopes* fungus. In addition, virus elimination, virus particles transfection, and biological comparison indicated that ClPV1 could lead to hypovirulence of the *C. liriopes* fungus.

Members of the family *Partitiviridae* are generally considered latent on their hosts (Ghabrial et al., 2015), even though some of them cause morphological alterations (Magae and Sunagawa, 2010; Bhatti et al., 2011; Jiang et al., 2019), hypovirulence (Potgieter et al., 2013; Xie and Jiang, 2014; Zheng et al., 2014), or hypervirulence (Lau et al., 2018). To our knowledge, there have been no reports of hypovirulence caused by a partitivirus or even mycovirus infection in the pathogenic fungus *C. liriopes.* In our study, ClPV1 could induce a reduced pathogenicity phenotype, according to virus elimination and biological analysis. In addition, we transfected the purified virus particles of ClPV1 into a virus-free, wild type *C. liriopes* strain SJM3-2 by a PEG-mediated protoplast transfection method. An isogenic fungal strain of SJM3-2, SJM3-2-PT5 that was infected by ClPV1, also showed a reduced virulence when compared to SJM3-2, indicating that ClPV1 could induce hypovirulence on the *C. liriopes* fungus.

The *C. liriopes* strains Cl-B-2 and SJM3-2 were originally isolated from *P. polyphylla* and *P. terminalis* plants, respectively. However, these plants were not easy to grow and to observe in virulence assays. We previously showed that *C. liriopes* could infect apples by artificial inoculation experiment. Thus, we selected the apple fruits for virulence assay in this study. It is common for a fungus to be infected by multiple interacting viruses. The hypovirulent phenotype might be found in some fungal strains that were co-infected by multiple viruses (Wang et al., 2014). Besides, some partitiviruses might induce host hypovirulence only if other co-infected mycoviruses were presented (Sasaki et al., 2016; Arjona-López et al., 2020), which indicated that some partitiviruses might interact with other mycoviruses and contribute to host hypovirulence. In our study, we conducted a high-throughput sequencing of strain SJM-2 before virus transfection and found no viral sequence in this strain (PRJNA727277), thus excluding the possible interference by other viruses that cannot be detected by conventional methods in the *C. liriopes* fungus. Moreover, we used virus elimination and transfection to fulfill the Koch’s postulates, therefore, we confirmed that the hypovirulent phenotype of the *C. liriopes* strains was caused only by virus ClPV1.

Although we have evidenced that ClPV1 could causes hypovirulence in the *C. liriopes* fungus. However, the experimental host range of ClPV1, which might be associated with the biological control potential, should also be explored. In the other hand, the interaction system of ClPV1-*C. liriopes* could also be used for elucidating the pathogenic mechanism of *C. liriopes.* In order to clarify the mechanism of hypovirulence caused by ClPV1 infection or to screen the key virulent associated genes in *C. liriopes*, more further studies are needed, including the transcriptome and proteome analysis of isogenic virus-infected and virus-free *C. liriopes* strains as well as the gene genetic transformation experiments.

## Data Availability Statement

The datasets presented in this study can be found in online repositories. The names of the repository/repositories and accession number(s) can be found below: https://www.ncbi.nlm.nih.gov/genbank/, MW291533; https://www.ncbi.nlm.nih.gov/genbank/, MW291532.

## Author Contributions

JZ and XL conceived and designed the experiments. JZZ, JG, and ZH performed the experiments. XZ analyzed the data. JZ, JZZ, JG, and ZH wrote the manuscript. All authors contributed to the article and approved the submitted version.

## Conflict of Interest

The authors declare that the research was conducted in the absence of any commercial or financial relationships that could be construed as a potential conflict of interest.
