# Supplementary material for: A Novel Partitivirus That Confer Hypovirulence to the Plant Pathogenic Fungus Colletotrichum liriopes
Source: Front Microbiol. 2021 Jun 22;12:653809. doi: 10.3389/fmicb.2021.653809 (PMC8262616; doi:10.3389/fmicb.2021.653809)
Supplement: Supplementary file 1 [file Data_Sheet_1.docx]

Fig.S1

**FIGURE S1** Phylogenetic analysis of the CP of ClPV1. The phylogenic tree was generated using the Neighbor-joining method in MEGA 6, with a 1000-replicate bootstrap search. ClPV1 was denoted in the phylogenetic tree. The scale bars indicated the estimated number of substitutions per 100 amino acids.

Fig.S2


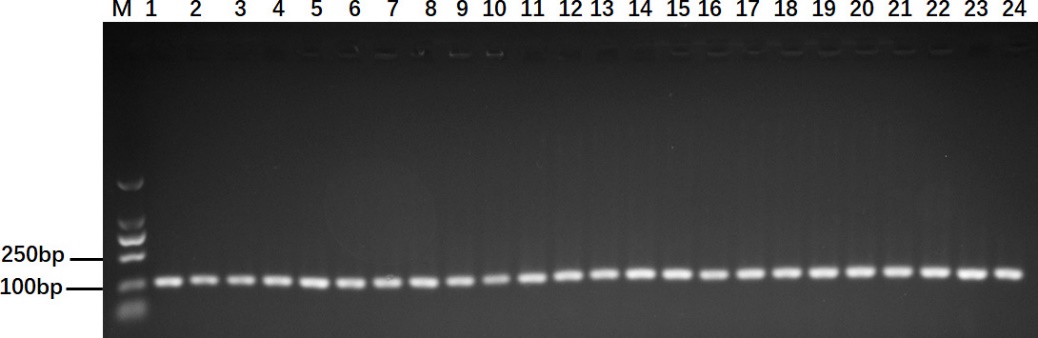


**FIGURE S2** Transmission of ClPV1 by conidium. The virus was detected in some representative single-spore strains by RT-PCR using specific primer pair designed based on the ClPV1 RdRp encoding sequence. M: Marker; 1 to 24: different single-spore strains derived from Cl-B-2.

Fig. S3


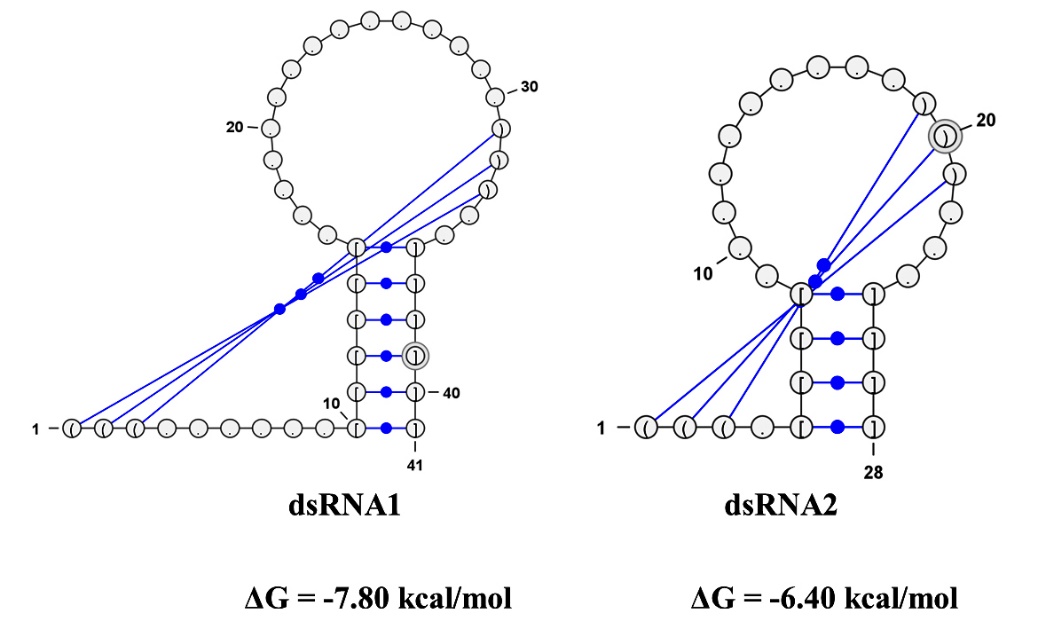


**FIGURE S3** Predicted secondary structure of the 5' UTRs of dsRNA1 and dsRNA2 in ClPV1. Stem-loop structure could be predicted in the 5' termini of dsRNA1 and dsRNA2, with the ΔG values of -7.8 kcal/mol and -6.4 kcal/mol, respectively.
